# Regulating Intestinal Microbiota in the Prevention and Treatment of Alcohol-Related Liver Disease

**DOI:** 10.1155/2020/6629196

**Published:** 2020-12-17

**Authors:** Xin Chi, Calvin Q. Pan, Shunai Liu, Danying Cheng, Ziwen Cao, Huichun Xing

**Affiliations:** ^1^Center of Liver Diseases, Beijing Ditan Hospital, Capital Medical University, Beijing, China; ^2^Beijing Key Laboratory of Emerging Infectious Diseases, Institute of Infectious Disease, Beijing Ditan Hospital, Capital Medical University, Beijing, China; ^3^Division of Gastroenterology and Hepatology, NYU Langone Health, New York University School of Medicine, New York, NY, USA; ^4^Department of Immunology, Capital Medical University, Beijing, China

## Abstract

When alcohol-related liver disease occurs, the number and composition ratio of intestinal microorganisms will accordingly change. The alcohol-induced changes in the intestinal microbiota play a pivotal role in the process of developing the alcohol-related liver disease through the translocation of microbial products due to increased intestinal permeability. In recent years, therapeutic interventions with a concentration on regulating intestinal microbiota have been conducted for patients with alcohol-related liver disease. We aimed to provide a critical review and updates on the prevention and treatment of alcohol-related liver disease through regulating intestinal microbiota. A literature search was performed on the PubMed database for studies published in English about the therapeutic intervention with microbiota using animal models and patients with alcohol-related liver disease (1/2010–4/2020). The accumulating pieces of evidence suggest that the therapeutic use of probiotics, prebiotics, antibiotics, phages, or fecal microbial transplantation may have several influences on alcohol-related liver disease patients. Emergent data unveiled that these interventions can further regulate the composition of intestinal microbiota, minimize the negative impact of microbiota on the liver, and prevent disease progression from mild to severe alcoholic hepatitis, fibrosis, cirrhosis, or even liver cancer. The current review provides updates on the advances of therapeutic interventions with the effects of regulating intestinal microbiota on patients who have alcohol-related liver disease. In addition, the data gaps and research directions on further exploration of the role of intestinal microbiota for the management of the alcohol-related liver disease are also discussed.

## 1. Introduction

Alcohol-related liver disease (ALD) is a chronic progressive disease caused by alcohol, ranging from hepatic steatosis to a more advanced stage, including alcoholic hepatitis (AH), liver fibrosis, ALD cirrhosis (AC), and hepatocellular carcinoma. ALD is a major cause of liver disease worldwide, both on its own and as a cofactor in the progression of chronic viral hepatitis, nonalcoholic fatty liver disease (NAFLD), iron overload, and other liver diseases [1]. According to the World Health Organization report on alcohol and health, alcohol causes 50% of liver cirrhosis and 10% of liver cancer deaths [2]. Long-term heavy drinking is the main pathogenic factor of ALD. In addition, excessive intake of alcohol may cause unpredictable pathological change or injury to the liver, intestine, brain, and other organs [1]. A large amount of alcohol drinking can lead to massive necrosis of liver cells and increase the risk of liver failure. In addition to the direct effects of alcohol on liver injury, other indirect effects of alcohol on liver injury have also been further explored. Recently, many studies have concentrated on the relationship between the liver and gut microbiota [3]. The liver is an organ that is closely associated with the intestine, and it is located at the intersection of intestinal circulation and portal blood flow of peripheral organs. Nutrients and bacterial compounds enter the liver through the portal circulation, maintaining the body balance under normal physiological conditions. The gut-liver axis connects the gut to the liver and represents a close functional and bidirectional communication between the gut and its microbiota and the liver. As a result, the dynamic changes of intestinal microorganisms play a pivotal role in the development of liver diseases through the integration of signals generated by dietary, genetic, and environmental factors [4].

In ALD, alcohol exposure can cause overgrowth of intestinal Gram-negative bacteria [5]. The change of intestinal microflora showed that the proportion of *Proteus* and *Fusobacteria* increased and the species of *Bacteroides* and *Lactobacillus* decreased [6]. In addition, the diversity of intestinal fungal microbiota in ALD decreased, and the growth of *Candida* was remarkable [7]. Antifungal therapy can reduce intestinal fungal overgrowth, reduce *β*-glucan, and improve liver injury induced by ethanol [7]. Bajaj et al. [8] compared patients with alcoholic cirrhosis with cirrhotic patients without a history of alcohol use. They found a significantly lower cirrhosis dysbiosis ratio (CDR) in the alcoholic group, which was defined as the ratio of autochthonous to nonautochthonous taxa. In addition, there was not only a significant increase in the relative abundance of *Enterobacteriaceae* and *Halomonadaceae* as well as the level of endotoxemia but also a remarkable decrease in the number of *Spirillaceae, Oncococcaceae,* and *Clostridium* in the alcoholic group [8]. Thus, chronic alcohol ingestion results in both quantitative changes of the intestinal microflora and enteric dysbiosis, which is known as a condition of unbalanced microflora and the loss of the symbiotic relationship between the host and microbiome.

**Figure 1:**
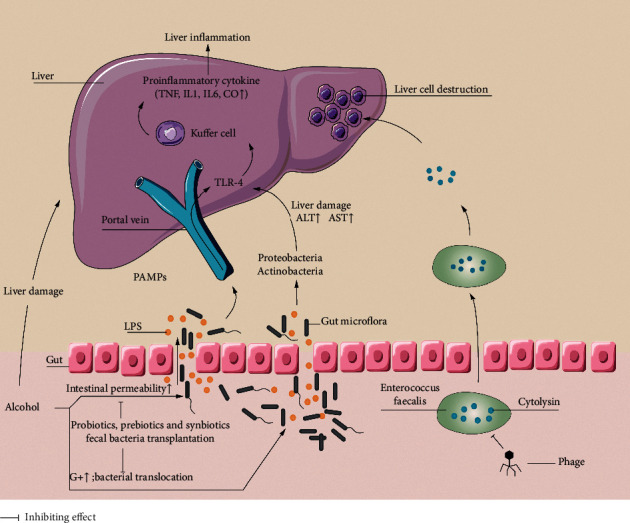
Mechanism of interaction between alcohol-related liver disease and intestinal microbiota. In alcohol-related liver disease, after long-term exposure to alcohol, the liver is directly damaged by alcohol; the expression of tight junction protein in intestinal epithelial cells decreases, the intestinal permeability increases, and the intestinal barrier function is damaged, accompanied by the higher level of endotoxemia. Intestinal microbiota disorders, intestinal microorganisms, bacterial metabolites, toxins, etc., are transferred from the intestinal tract to the liver, resulting in liver damage. LPS produced by *Enterogenous* bacteria binds to TLR-4, mediates the activation of liver Kupffer cells. The activated Kupffer cells release a large number of proinflammatory cytokines, induce liver inflammation. Cytolysin produced by *Enterococcus faecalis* has a dissolving effect on liver cells. Phages can target *Enterococcus faecalis* and reduce the liver damage. Probiotics, prebiotics, and fecal bacteria transplantation etc., can improve the liver function of patients with alcohol-related liver disease by regulating intestinal microbiota and improving intestinal mucosal permeability. LPS: lipopolysaccharide; G+: Gram-positive bacteria; TLR-4: toll-like receptor 4; PBMCs: peripheral blood mononuclear cells; IL-1: interleukin-1; TNF: tumor necrosis factor; PAMP: pathogen-associated molecular pattern; CO: carbon monoxide; ALT: alanine transaminase; AST: aspartate transaminase.

Another very important pathogenesis of ALD is the translocation of intestinal microbiota and microbial products, resulting from an increased intestinal permeability (Figure 1). Bjarnason et al. [9] showed that alcoholic patients had significantly higher permeability of the small intestine than control patients when the 51Cr-ethylenediaminetetraacetic acid (EDTA) absorption test was performed. Patients with ALD may suffer from the “leakage” of intestinal bacterial metabolites, including bacterial DNA, bile acid, peptidoglycan, and flagellin. Subsequently, the bacteria and its metabolites were transferred from the intestinal lumens to the liver, which led to liver injury [10, 11]. It is also found that alcohol intake not only aggravates liver injury in patients with chronic hepatitis C (*n* = 139) or with chronic hepatitis B (*n* = 61) [12] but also affect intestinal microbiota in patients with HBV-related chronic liver disease [13]. At the family level, alcohol intake induced *Lachnospiraceae* family and *Veillonellaceae* family decreased, and *Bacteriodaceae* family increased in Child-Pugh grade A (CPA) patients. At the genus level, alcohol intake increased *Bacteroides* (CPA and Child-Pugh grad C) and *Megamonas* (CPA) and *Veillonella* (Child-Pugh grade B) level, respectively. Because the change of intestinal microbiota is associated with the degree of liver damage, the susceptibility of liver injury in patients with ALD may be reduced by modifying the composition or balance of intestinal microbiota [14]. Therefore, regulation of intestinal microecology plays a potential role in the treatment of patients with ALD. In the current review, we aimed to present and summarize the advances made recently on the microbiota-related models for the prevention and treatment of ALD (not with HBV or HCV infection), involving probiotics, prebiotics, antibiotics, phages, and fecal microbial transplantation (FMT).

## 2. Data Searches and Synthesis

A literature search was performed on the PubMed database for studies published in English about the therapeutic intervention with microbiota on animal models and patients with ALD (1/2010–4/2020). Keywords of alcohol-related liver disease/ALD; intestinal microbiota; treatment; phage; fecal bacteria transplantation; treatment of alcohol diseases; and clinical outcomes were used to identify the relevant publications. The literatures involved in viral hepatitis (such as HBV and HCV infection), autoimmune liver diseases, and drug-induced liver injury were excluded. The literature search was performed by the first author (Chi X), and a list of potential eligible publications was generated by screening the titles and abstracts. Each publication on the list of preselected studies was further reviewed by the two authors (Xing HC and Pan CQ) independently to determine if the study fulfilled the relevant criteria. The authors (Chi X, Liu SA, Cheng DY, and Cao ZW) performed the extraction of data from the selected publications independently. The data that were collected from the selected studies included the date of publication, study design, study materials or patients, the sample size, study period and methods, treatment regimens, and study conclusions with the authors' recommendations. Lastly, the data were integrated under the direction of Xing HC and Pan CQ.

## 3. The Effects of Probiotics, Prebiotics, and Synbiotics

Probiotics are defined as a kind of active microorganisms that are composed of single or mixed cultures of microorganisms, which can improve the characteristics of existing intestinal microbiota, prevent bacterial translocation, inhibit the formation of endotoxin, promote the formation of an anti-inflammatory environment, and maintain the integrity of intestinal barrier [5]. The role of probiotics in ALD has been explored in animal models. Bang et al. [15] performed a study on 60 C57BL/6 mice and randomized them equally into 6 feeding groups for 10 weeks: normal diet, alcohol, control, alcohol + Korean red ginseng, alcohol + urushiol, and alcohol + probiotics. The alcohol was administered via a Lieber-DeCarli liquid diet containing 10% alcohol. The levels of toll-like receptor 4 (TLR-4), proinflammatory cytokines, and histology, as well as the results of liver function tests, were measured and compared. They observed that the TLR-4 level was significantly lower in the probiotics groups than that in the alcohol group (0.33 ± 0.07 ng/mL vs. 0.88 ± 0.31 ng/mL; *P* < 0.05). The interleukin-1*β* (IL-1*β*) level in liver tissues was also decreased among the probiotics groups, which suggested the effects of reducing inflammation in the liver with probiotics.

TLR-4 is a pattern recognition receptor and is expressed in several cells in the liver. TLR-4 also serves as a transmembrane protein and a lipopolysaccharide (LPS) sensor, the LPS and LPS-related complex circulated from the intestine to the liver due to the increased intestinal permeability in ALD. After binding to the TLR-4, LPS can activate Kupffer cells and peripheral blood mononuclear cells (PBMCs) through the nuclear factor-kappa B (NF-*κ*B) pathway and IL6/STAT3 signaling pathway. Subsequently, the activated Kupffer cells and PBMCs could release a large amount of proinflammatory, antiviral, and antibacterial cytokines [16]. Therefore, the decrease of the TLR-4 level in probiotics-treated patients led to the reduction of liver inflammation [17]. In addition to the aforementioned investigations, the main results of other animal experiments are presented in Tables 1 and 2, and they all pointed in the same direction and came to similar conclusions.

Advancing from the animal models, several clinical trials have investigated the therapeutic effects of probiotics on ALD patients. Han et al. [25] studied the relationship between probiotics and liver injury in patients with AH. They noted that AH patients (*n* = 60) had a significant reduction in the number of intestinal *E. coli* and the level of LPS after receiving the combined treatment of *Lactobacillus subtilis* and *Streptococcus faecium*, followed by the improvement of liver functions. In another study [24], patients with alcoholic cirrhosis (*n* = 12), who were treated with *Lactobacillus casei Shirota* (6.5 × 10^9^ CFU) three times daily for 4 weeks, were compared with the cirrhosis patients without undergoing probiotic therapy (*n* = 8). In patients who underwent probiotic therapy, the TLR-4 level was decreased and the neutrophil phagocytosis was restored. In addition, in patients who were treated with probiotics, several prespecific measurements were markedly improved, which included the composition of intestinal microbiota, reduction of the serum endotoxin levels, and improvement of intestinal barrier function.

Prebiotics are substances that contribute to the growth and activity of specific microorganisms in the gastrointestinal tract and are beneficial to the host [30]. They are not easily metabolized and digested by pancreatic and intestinal enzymes [22]. Most importantly, prebiotics can selectively stimulate the growth of beneficial bacteria (e.g., *Bifidobacteria* and *Lactobacilli*) [31]. A previous study demonstrated that the relative abundance of *Bacteroides* was increased in mice fed with alcohol. However, their liver injury was decreased when those mice were treated with pectin [23]. As another prebiotic, lactulose was found to promote the metabolism of colonic bacteria and produce acetic acid as well as lactic acid, which could decrease the intraluminal pH in the colon. This then inhibited the growth of urease-producing bacteria and increased the number of probiotic *Lactobacilli* [26]. In recent years, a number of studies have pointed out several therapeutic effects of prebiotics on mice, which included reducing the levels of oxidative stress markers and inflammatory markers, upregulation of the expression of connexin in the intestinal wall, as well as decreasing the plasma level of endotoxin [31].

Synbiotics are the combination of probiotics with prebiotics and may have several synergistic effects, which are highlighted as follows: (1) providing a selective medium for the growth of a probiotic strain, as well as reducing concentrations of undesirable metabolites; (2) modulating the metabolic activity in the intestine and developing beneficial microbiota; (3) inhibiting potential pathogens present in the gastrointestinal tract and increasing the tolerance to environmental conditions, such as modifying oxygenation process and pH in the intestine lumen [32–34]. Based on the aforementioned synergistic effects, a variety of symbiotic formulas have been recently presented. For example, FloraGuard® is a kind of composite powder rich in prebiotics and *Lactobacillus acidophilus*, *Lactobacillus bulgaricus, Bifidobacterium, Bifidobacterium longum*, and *Streptococcus thermophilus*, and it can significantly improve the intestinal ecosystem of rats by increasing the probiotic population (*Bifidobacterium* and *Lactobacillus*) and activity of some digestive enzymes [35]. In another study, patients with cirrhosis and mild hepatic encephalopathy were treated with Synbiotic 2000. The Child-Pugh level was improved in 47% of patients, which was higher than that in patients treated with fermentable fiber (29%) or placebo (8%) [26]. The combination of prebiotics and probiotics may thus have potential therapeutic influences on ALD patients.

## 4. The Role of Bacteriophage

Bacteriophage is the only virus that can specifically infect and kill bacteria. There are up to 10^15^ phage particles in the human intestine [36], which have great biodiversity in nature. Phages are highly specific to the bacterial subtypes and can selectively infect specific bacteria, the effect of which is equivalent to knocking down specific bacteria (close to the reduction effect of bacterial deletion) [37]. Owing to its diverse advantages compared with antibiotic therapy, the engineered phages are less likely to provoke resistance in bacteria and may assist to overcome antibiotic resistance. It is noteworthy that phage is used to treat antibiotic-resistant bacterial infection [38]. Additionally, whether phages are used on their own or combined with antibiotics, they are still such a promising agent to replace with antibiotics [39].

In a mouse model, Hsu Bryan et al. [40] selected 10 kinds of bacteria (representing the main phylum of human intestinal microbiota), including *Firmicutes* (*Clostridium sporogenes* and *Enterococcus faecalis*), *Bacteroidetes* (*Bacteroides fragilis, Bacteroides ovatus, Bacteroides vulgatus,* and *Parabacteroides distasonis*), *Proteobacteria* (*Klebsiella oxytoca, Proteus mirabilis*, and *Escherichia coli Nissle 1917*), and Verrucomicrobia (*Akkermansia muciniphila*). The investigators inoculated mice with these bacteria (2 × 10^6^ CFU for each species of *Akkermansia muciniphila* and *Proteobacteria* and 2 × 10^7^ CFU for each other) on day 0. Subsequently, the mice were given different phages on two different days, which were the combinations of T4 with F1 (targeted at *E. coli* and *Clostridium sporogenes*, respectively) on day 16; also, the mixture of B40-8 with VD13 (targeted at *Bacteroides fragilis* and *Enterococcus faecalis*, respectively, the amount of each phage was 2 × 10^6^ PFU) was undertaken on day 30. The fecal samples were collected to analyze the changes in bacterial species and metabolites. They observed that the number of *Clostridium sporogenes* and *Escherichia coli* significantly decreased in the feces after the first group of phages were given, while the number of *Bacteroides fragilis* and *Enterococcus faecalis* had increased due to the absence of the targeting phages. However, after the administration of the second group of phages, *Bacteroides fragilis* and *Enterococcus faecalis* were simultaneously knocked down. These two species of bacteria may have either inhibitory or promotion effects on *Akkermansia muciniphila, Bacteroides ovatus, common Bacteroides*, and *Proteus mirabilis*, respectively. In the context of bacterial interaction, when two bacteria were simultaneously knocked down, their effect on the whole microbial population was insignificant.

Besides their effects on bacteria, phages can also regulate the metabolism of specific substance-related bacteria. Hsu et al. [37] demonstrated that the levels of tryptamine (a neurotransmitter metabolite) were decreased by 10, 17, and 2 folds in the days 0.3, 2, and 13 after the first administration of phages, respectively, which reflected that the number of corresponding *Clostridium* decreased by 840, 4, and 4 times at the same time points. During the same period, the levels of serine and threonine, two representative amino acids, were increased. Additionally, the levels of different bile salt components in the feces were markedly changed, which included the increased levels of bile salt sulfate and the decreased levels of conjugated bile salt. Furthermore, after the administration of the second group of phages, the levels of tyramine were decreased by 4, 2.7, and 4 folds on days 0.3, 2, and 13, respectively. These changes reflected the decreased number of the corresponding *Enterococcus faecalis* by 1.3, 9, and 42 folds at the same time points, respectively. In addition, other compounds related to the microbial metabolism were also widely affected in that experiment.

In the disease-specific investigations, phages have been used to regulate intestinal microbiota and treat intestinal-related diseases, such as Crohn's disease, ulcerative colitis, and irritable bowel syndrome [41]. Abnormal proliferation of adherent invasive *Escherichia coli* (AIEC) in the ileum mucosa of patients with Crohn's disease (CD) was assessed [42]. Galtier et al. [42] assessed the potential of bacteriophages, viruses infecting bacteria, to decrease the levels of AIEC bacteria colonization in the intestinal mucosa. In their experiment, the 10-week FVB/N CEABAC10 transgenic mice (with high levels of expression of human CEACAMs, LF82 efficiently binds to epithelial cells) were colonized with LF82 by oral challenge with 1 × 10^9^ LF82 bacteria. On the next day, three phage mixtures (LF82_P2, LF82_P6, and LF82_P8 3 × 10^7^ PFU each) were administered orally twice a day with 7 hours apart by oral gavage. One or four days after administration, the concentration of AIEC strain LF82 in feces of the phage-treated group decreased significantly. They further confirmed that the preventive use of phages can alleviate the colitis symptoms caused by dextran sodium sulfate, and three kinds of phages can be replicated in all intestinal segments in vitro. The experiment suggested that phages could potentially treat CD by regulating the balance of intestinal microbiota.

Recently, the disease-specific investigations with phage have been extended to ALD. Duan et al. [43] cultured *Enterococcus faecalis* strains from fecal samples of patients with AH and isolated phages against *Enterococcus faecalis*-positive for cytolysin (a bacterial exotoxin produced by *Enterococcus faecalis* that dissolves Gram-positive bacteria and eukaryotic cells) (Figure 1) from sewage. In their experiment, mice that were inoculated with human fecal bacteria into the intestine through feeding were fed with chronic ethanol to establish a model. When compared to the control group without the treatment of phages targeting any bacteria, the experiment group which received phages targeting at *Enterococcus faecalis* and its related production of cytolysin had a significant decrease in the severity of the liver damage, inflammation level, and fatty degeneration based on the prespecified parameters. Such therapeutic effects of phages on the liver were considered to be secondary to the reduction of cytolysin levels in the liver. That study shed a light on the phage therapy for ALD, and further clinical studies are ongoing to clarify its efficacy for patients with ALD. Despite the promising results of phages on ALD, phage therapy has some limitations. For instance, because the structure of enterococcal polysaccharide antigen (EPA) can affect phage's ability to infect *Enterococcus faecalis*, the mutation of the EPA gene with subsequent changes in the EPA structures leads to phage resistance and prevents phage from infecting the bacteria. However, the EPA mutation may result in regaining bacterial susceptibility to antibiotics, which may provide an opportunity for the combination of antibiotics and phages or sequencing therapy as antibiotic remains the mainstream therapy for preventing the overgrowth of harmful bacteria and improves the maladjusted intestinal microbiota [44].

Although the therapeutic role of intestinal phages in human diseases has been explored and in progress, few data are available on the interaction between the phages and intestinal bacteria. The complex phage community is one of the biggest knowledge gaps in understanding the composition of human microorganism [45]. Recently, Shkoporov et al. [46] studied fecal viruses in healthy adults and found that there were two kinds of stable (>1 year) and dominant fecal viruses in the human intestine, which are crAss-like phages (highly virulent) and microviridae phages. When bacteria are infected with phages, the clustered regularly interspaced short palindromic repeats (CRISPR), as a family of DNA sequences, can be found in the genomes of bacteria, which are used to detect and destroy DNA from similar bacteriophages during subsequent infections. Shkoporov et al. demonstrated that CRISPR-based host prediction revealed high temporal stability, individual specificity, and correlation with the bacterial microbiome, which further highlighted correlations between these stable viral communities and highly predominant gut bacterial taxa (e.g., *Bacteroides*, *Prevotella*, and *Faecalibacterium*). Their data on the structure of the human gut virome may set the stage for hypothesis-driven research on the therapeutic of phages on disease specified clinical scenario. At present, numerous clinical studies are aiming at phage therapeutic intervention, including cocktail made by mixing a variety of phages for diseases at different stages, and the status of antibiotic resistance [47, 48]. In addition, the binding sites of phage on bacteria have been explored to eliminate specific bacteria and maintain healthy microbiota [49].

## 5. The Efficacy and Safety of FMT

FMT is suggested as an efficacious therapeutic strategy for restoring intestinal microbial balance and thus for treating a disease associated with alteration of gut microbiota. FMT consists of the administration of fresh or frozen fecal microorganisms from a healthy donor into the intestinal tract of diseased patients. FMT is a conventional and effective method for the treatment of *Clostridium difficile* infection (CDI) [50]. There are also some potential therapeutic indications, such as metabolic syndrome, irritable bowel syndrome, primary sclerosing cholangitis, cancer, and other diseases related to intestinal microbiota disorder. At present, some scholars have concentrated on the treatment of ALD patients with FMT [50].

In one study, mice with alcoholic fatty liver were treated with FMT. The results showed that the intestinal microbiota of mice treated with FMT was recovered, and the degree of liver steatosis and inflammation was decreased [23]. Several studies have confirmed that FMT plays a significant therapeutic role in severe alcoholic hepatitis (SAH). In a study performed by Philips et al., on patients with SAH who were not able to tolerate corticosteroid therapy, 8 patients with SAH were daily administrated with fecal microbiota through the nasoduodenal tube for 7 days. Meanwhile, 18 patients with SAH served as control and were treated with a standard operation sheet (SOS). Compared with the control group, patients in the group with FMT had a significant reduction in the severity of liver disease and the improvement of 1-year survival (33.3% vs. 87.5%; *P*=0.018) [51]. In a subsequent clinical study carried out by Philips et al. SAH patients were divided into 4 treatment groups (8 cases treated with glucocorticoid, 17 cases with simple nutrition support, 10 cases with pentoxifylline, and 26 cases with FMT) [52]. At the end of one month of the therapy, the survival rates of the aforementioned 4 groups were 63%, 47%, 40%, and 75% (*P*=0.179), respectively. At the end of 3 months of the treatment, the corresponding survival rates were 38%, 29%, 30%, and 75% for these 4 groups (*P*=0.036), respectively. These results indicated that FMT could improve the survival rates of SAH patients. Therefore, the study strongly supported FMT as one of the treatment options for SAH, and the authors contributed the following effects of FMT for the improvement of survival rates: relative abundance of intestinal microbiota and enhance the levels of metabolites, regulation on the inflammatory response, and reduction of oxidative stress.

The FMT therapy for end-stage liver disease from ALD or cirrhosis from other etiologies has been also investigated recently. In a series of clinical controlled trials, Bajaj et al. [53–55] treated patients with cirrhosis and recurrent hepatic encephalopathy using antibiotics, and then the oral-fecal microbial capsule was taken. Compared with the standard operation sheet (SOS) group, patients in the FMT-treated group experienced the following significant benefits: the disturbance of microbiota was restored; the diversity and quantity of beneficial bacteria were increased, which included *Ruminococcaceae* and *Bifidobacterium*; the short-term cognitive function was improved, and the rate of recurrence and readmission of hepatic encephalopathy was decreased. The beneficial effect of FMT on cognitive function may be due to the decrease of neuroinflammation and the improvement of microglia activation, which was confirmed by Liu et al. [56]. In their experiment, germ-free (GF) mice were colonized with cirrhotic patients' stool obtained before and after FMT for making a comparison. The frontal cortex, liver, and small/large intestines of mice were collected to analyze the cortical inflammation, synaptic plasticity, and gamma-aminobutyric acid (GABA) signaling, as well as liver inflammation and intestinal 16s ribosomal RNA microbiota sequencing. Their results demonstrated that fecal microbial colonization from patients with cirrhosis results in higher degrees of neuroinflammation and activation of GABAergic neurons in mice. The reduction in neuroinflammation by using samples from post-FMT patients to colonize mice was a direct effect of fecal microbiota and independent of active liver inflammation or injury.

Although FMT is a promising treatment modality, emerging evidence supports its clinical application for patients with ALD and other liver diseases, challenges and the safety-based concerns exist. Recently, the European expert consensus on the clinical application of FMT has been published and addresses several clinical relevant issues, including the indications of FMT, the selection of donors, the preparation of fecal materials, the use of FMT, and clinical management of FMT recipients [50]. Further efforts for implementation of FMT in clinical practice should include the following aspects: establishing standardized fecal banks; establishing a database for monitoring the safety and tolerance of FMT; and facilitating patients' access to FMT [57].

## 6. Other Microbiological Methods

Traditionally, antibiotics have been used to eliminate local or systemic infection and are also used in the treatment of liver cirrhosis to reduce and avoid complications, such as spontaneous bacterial peritonitis and hepatic encephalopathy [17]. In patients with hepatic encephalopathy, the nonabsorbable antibiotic rifaximin is the first line of treatment due to its effects on inhibiting bacterial RNA by combining with *β* subunit of DNA-dependent RNA polymerase. Rifaximin has also shown a broad-spectrum antibacterial activity [58]. In a randomized controlled trial (RCT) that provided 8 weeks of treatment with rifaximin (550 mg BID) versus placebo, 20 patients with liver cirrhosis and mild hepatic encephalopathy exhibited significant changes in bacterial metabolites, decreased serum endotoxin level, significantly increased levels of fatty acids, and improved brain cognitive function [59]. Nevertheless, the effect of antibiotics is not relevant in the course of chronic therapy in patients. Yaq-001 is a newly synthesized nonabsorbable carbon with a high capacity of adsorbing bacterial toxins. Another new development is a therapy by using carbon with a uniquely tailored porosity, conferring a high absorptive capacity for gut-derived bacterial metabolites and toxins relevant to pathogenesis in liver disease. In a preclinical study, Yaq-001 selectively modulates the stool microbiome and its function, which is associated with the restoration of immune function and inflammasome activation [60]. In addition, a randomized, double-blind, and placebo-controlled trial is investigating the safety and tolerability of oral yaq-001 in patients with decompensated cirrhosis, which will clarify the clinical implication of yaq-001 (https://clinicaltrials.gov/ct2/show/NCT03202498).

Regarding synthetic live bacterial therapy, synthetic engineering probiotics can produce a large number of low molecular acids, hydrogen peroxide, and antibacterial active peptides. The therapy also reduces toxic substances and inhibits the growth and reproduction of harmful bacteria. In a preclinical study conducted by Singh et al. pyrroloquinoline quinone was exhibited to improve ethanol-induced liver injury in rats. The probiotic *Escherichia coli Nissle 1917* (EcN) was modified to a secret pyrroloquinoline quinone. They found that, when male Charles-Foster rats were treated with EcN for 10 weeks, the levels of oxidative stress in the liver were decreased [61].

Recent investigations have been conducted on the expression of FXR to reduce the level of harmful microbial metabolites and protect the liver from injury [62–64]. FXR agonists (GW4064) can reduce the production of bile acids, inhibit the overgrowth of intestinal flora, protect the integrity of intestinal mucosa, and reduce the degree of liver inflammation and steatosis [65, 66]. Other researches have concentrated on the gut-liver axis, and LPS may activate the TLR-4 pathway and mediate the liver injury caused by ALD through a series of cascade reactions. In TLR-4 knockout and mutant ALD mice, scholars observed the improvement of liver inflammation and steatosis and suggested that methods of knocking out TLR-4 and regulating microbial metabolic pathways are promising to prevent and treat ALD [67].

## 7. Summary and Future Research Directions

In conclusion, intestinal microbiota plays a significant role in the occurrence and development of ALD. Regulating intestinal microbiota can be a prospective strategy for the prevention and treatment of ALD. Advances have been made to explore therapeutic interventions through manipulating or regulating intestinal microbiota and their metabolites. Studies demonstrated that probiotics, prebiotics, antibiotics, phages, and FMT can selectively adjust intestinal microorganisms. All of these have also great potential to prevent and treat ALD effectively by rebuilding microbial balance in the intestinal microbiota, although the effectiveness and rationality of the new microbiological treatment still require to be further explored. Future research should be conducted to design a broad-spectrum bacteriophage therapy to prevent and treat diseases related to microbiota imbalance in patients with ALD because of the limited therapeutic options that existed for the disease. The endpoint assessment of ALD in clinical trials related to regulating intestinal microbiota and their metabolites are needed to be standardized with the collaboration between the entities of research/drug development and local drug administration authorities. Eventually, the barriers to implementation of treatment methods, such as FMT, should be addressed, which include financial difficulty, patients' acceptance, and cultural resistance.

## Figures and Tables

**Table 1:** Animal experimental studies on the treatment of ALD with probiotics, prebiotics, and synbiotics.

Study	Object	Types of drug	Outcomes
Forsyth et al. [18]	Alcoholic steatohepatitis male Sprague-Dawley rats	*Lactobacillus rhamnosus* (LGG)	Liver steatosis severity reduced
Chang et al. [19]	Male wild type rats with acute alcohol-related liver disease	VSL#3 (a mixture of probiotics such as *Lactobacillus acidophilus* and *Lactobacillus plantarum*)	Increased intestinal permeability (decreased plasma endotoxin and TNF*α* levels)
Bang et al. [15]	Alcohol-related liver disease C57BL/6 mice	*Lactobacillus rhamnosus* R0011 and *Lactobacillus acidophilus* R0052	Reduced inflammation of the liver (TLR4 expression decreased)
Grander et al. [20]	C57BL/6 mice with alcoholic steatohepatitis	Colistin	Increased gut barrier integrity (relative abundance of *A. muciniphila* and mucin is increased)
Tang et al. [21]	Male alcohol-related liver disease Sprague- Dawley rats	Oats (prebiotics)	Reduced oxidative stress (NOS, NO protein carbonylation, and nitrotyrosination) and increased gut barrier integrity (integrity of actin cytoskeleton and tight junction)
Yan et al. [22]	Alcohol-related liver disease mice	Fructooligosaccharide (FOS)	Improvement of the degree of liver inflammation (recovery of the level of antimicrobial protein Reg3g and reduction of intestinal bacterial overgrowth)
Ferrere et al. [23]	Mice fed by alcohol	Prebiotic pectin	Relative abundance of bacteroides was increased, improvement of the severity of steatosis, and reduction of inflammation in the liver

**Table 2:** Human experimental studies on the treatment of ALD with probiotics, prebiotics, and synbiotics.

Study	Object	Types of drug	Outcomes
Stadlbauer et al. [24]	Patients with alcoholic cirrhosis	*Lactobacillu casei*	TLR-4 expression decreased significantly, phagocytic function of neutrophils improved, and plasma endotoxin level reduced.
Han et al. [25]	Patients with AH	*Lactobacillus subtilis* and *Streptococcus faecium*	Decrease in the number of *E. coli*, LPS level, and proinflammatory cytokines
Fukui [26]	Patients with cirrhosis and mild hepatic encephalopathy	Synbiotic 2000® (4 freeze-dried nonurease-producing *Lactobacillus* and 4 fermentable fibers)	Child-Pugh level was improved
Lata et al. [27]	Patients with liver cirrhosis	*Escherichia coli Nissle*	The intestinal colonization of *E. coli* was significantly improved and the endotoxin level was decreased, Child-Pugh level was improved
Kwak et al. [28]	Patients with chronic liver disease	Six bacterial species were used: *Bifidobacterium bifidum*, *Bifidobacterium lactis*, *Bifidobacterium longum*, *Lactobacillus acidophilus*, *Lactobacillus rhamnosus*, *and Streptococcus thermophilus*	Intestinal bacterial overgrowth was alleviated, intestinal permeability and liver function did not improve significantly
Liu et al. [29]	Patients with minimal hepatic encephalopathy	Synbiotics (probiotics and fermentable fiber)	Child-Pugh level was improved and endotoxin level was decreased
